# Retraction: Role of Livelihood Capital in Reducing Climatic Vulnerability: Insights of Australian Wheat from 1990–2010

**DOI:** 10.1371/journal.pone.0275147

**Published:** 2022-09-20

**Authors:** The PLOS ONE Editors

Following the publication of this article [1], concerns were raised regarding similarity with a previously published study [2], as well as the methodology, results and conclusions discussed in this article [1]. The editorial team and several board members have re-evaluated the article and determined that the concerns listed below remain unresolved.

- The two articles [1, 2] use similar data sets to develop empirical measures of vulnerability, report temporal and spatial patterns, and assess impacts of capital indicators on vulnerability and/or adaptive capacity. Although the articles report somewhat different results regarding to the variables that are related to vulnerability and/or adaptive capacity, the board members comment that this article [1] is too similar to the earlier published study [2] to warrant a separate publication. Furthermore, a board member states that this article [1] does not present an adequate justification for the analysis of a similar, but older, data set in different ways, as no in-depth discussion for the differing methodologies was provided, nor does this article [1] discuss why the results of the two studies are different.
- This article [1] presents a less robust assessment of an older data set (2012), when a more up-to-date data set (2014) was available. One of the board members commented that the earlier article [2] presents a much more comprehensive assessment including more variables, and that the vulnerability relationships are implicitly evaluated in the earlier study [2] even if they are not calculated in the same way as presented in [1].
- Using the annual mean maximum temperature alone to calculate the wheat vulnerability index is a significant flaw in the methodology of this article, especially when other data relevant to wheat vulnerability and climate change exposure were available at the time of submission. A board member commented that if temperature were the only variable to be used, then it should at least include some index of temperature extremes such as heat wave duration, not an annual average. Furthermore, the board member points out that the wheat climatic vulnerability index (VI) cited [3] in this article [1] uses the better indicator of climate change exposure, season specific rainfall, as opposed to annual mean maximum temperature to calculate the index. The board member states that they could not see how a single factor could make for a good climate indicator, as there are many other influential factors that represent exposure of wheat to climate change, such as total volume and timing of rainfall, that do not always correlate to maximum temperature. The board member also questions whether annual maximum temperature is indeed used to measure drought.

The author disagreed with the editorial assessment. They commented that [1] and [2] differed in the data used, theoretical framework, hypotheses, methods, model, and conclusions. The author also stated that there are references that support measuring vulnerability with mean air temperature, and that they selected the 2012 dataset for this study but recognize that both the 2012 and 2014 datasets each have strengths and weaknesses. The *PLOS ONE* Editors concluded that the author’s statements did not resolve the methodological concerns, nor provide sufficient justification for publication of [1] in light of the closely related yet more robust study published earlier [2].

The *PLOS ONE* Editors retract this article [1] because per our editorial assessment it did not meet *PLOS ONE’s* Publication Criteria (#2, #3) [4]. We regret that the issues with this article were not identified and addressed prior to its publication.

The author did not agree with the retraction.
